# ChatGPT-4 Vision: a promising tool for diagnosing thyroid nodules

**DOI:** 10.3389/fmed.2025.1634976

**Published:** 2025-07-30

**Authors:** Dao-Rong Hong, Chun-Yan Huang, Huo-Hu Zhong, Guo-Rong Lyu

**Affiliations:** ^1^Department of Ultrasonography, The Second Affiliated Hospital of Fujian Medical University, Quanzhou, Fujian, China; ^2^Department of General Practice, The Second Affiliated Hospital of Fujian Medical University, Quanzhou, Fujian, China

**Keywords:** thyroid nodules, ultrasound, ChatGPT, diagnosis, pathology

## Abstract

**Objective:**

This study aims to evaluate the application of ChatGPT-4 Vision in the ultrasonic image analysis of thyroid nodules by comparing its diagnostic efficacy and consistency with those of sonographers.

**Methods:**

In this prospective study, conducted in real clinical scenarios, we included 124 patients with pathologically confirmed thyroid nodules who underwent ultrasound examinations at Fujian Medical University Affiliated Second Hospital. A physician, not involved in the study, collected three ultrasound images for each nodule: the maximum cross-sectional, maximum longitudinal, and the section best representing the nodular characteristics. The images were analyzed by the primed ChatGPT-4 Vision and classified according to the 2020 Chinese Guidelines for Ultrasound Malignancy Risk Stratification of Thyroid Nodules (C-TIRADS). Two sonographers with different qualifications (a resident physician and an attending physician) used the same images to classify the nodules according to the C-TIRADS guidelines. Using fine needle aspiration (FNA) biopsy or surgical pathology results as the gold standard, we compared the consistency and diagnostic efficacy of the primed ChatGPT-4 Vision with those of the sonographers.

**Results:**

(1) ChatGPT-4 Vision diagnosed thyroid nodules with a sensitivity of 86.2%, specificity of 60.0%, and an AUC of 0.731, which was comparable to the resident’s sensitivity of 85.1% (95% CI: 77.2–90.8%), specificity of 66.7% (95% CI: 53.7–77.7%), and AUC of 0.759 (*p* > 0.05), but lower than the attending physician’s sensitivity of 97.9% (95% CI: 93.2–99.5%), specificity of 80.0% (95% CI: 67.7–88.6%), and AUC of 0.889 (95% CI: 81.5–96.4%) (*p* < 0.05). (2) The primed ChatGPT-4 Vision demonstrated good consistency with the resident in thyroid nodule classification (Kappa value = 0.729), though its consistency with the pathological diagnosis was lower than that of the attending physician (Kappa values of 0.457 vs. 0.816, respectively).

**Conclusion:**

The primed ChatGPT-4 Vision demonstrates promising clinical utility in thyroid nodule risk stratification, achieving diagnostic performance comparable to resident physicians. Its ability to standardize image analysis aligns with precision medicine goals, offering a foundation for future integration with dynamic ultrasound modalities to enhance pathological correlation.

## 1 Background

With the rapid development of artificial intelligence (AI) technology, AI-assisted diagnosis is increasingly used in the medical field (1–4), particularly showing significant potential in imaging medicine (5, 6). Thyroid nodules are a common clinical condition with a prevalence rate of 19 to 68% (7), and differentiating benign from malignant nodules is crucial for choosing the appropriate clinical management. Ultrasound, known for being real-time, economical, and radiation-free, is currently the preferred method for examining thyroid nodules. However, ultrasound diagnosis depends on the experience of the physician interpreting the images, which is not only time-consuming but can also lead to inconsistent results due to individual differences in experience.

ChatGPT, as an advanced natural language processing tool, has begun to draw attention in the fields of medical image interpretation, drug synthesis, and education (8–12). Recent studies have focused on the application of ChatGPT-4 Vision in thyroid reporting and structured management (13), as well as comparisons of diagnostic performance among three major language models in thyroid nodule diagnosis (14). However, these studies are retrospective, based on textual analysis, and do not include comparisons with sonographers in real clinical settings.

While recent studies explored LLMs in thyroid nodule risk stratification (15, 16), this is the first prospective trial validating raw ultrasound image-based diagnosis by ChatGPT-4 Vision against pathological gold standard, with direct benchmarking against dual-tier clinician expertise (resident vs. attending physicians). Our design bridges critical gaps between text-based simulations and real-world image interpretation workflows.

Therefore, we prospectively explore the actual application value of ChatGPT-4 Vision in thyroid ultrasound image analysis and compare the diagnostic outcomes of ChatGPT-4 Vision with those of sonographers with varying qualifications, aiming to validate the potential of ChatGPT-4 Vision in diagnosing thyroid nodules and providing a practical foundation for the future expansion of medical AI technology in clinical applications.

## 2 Materials and methods

### 2.1 Clinical data and study subjects

This study included 124 patients with thyroid nodules who underwent ultrasound examinations and were pathologically confirmed at the Second Hospital Affiliated with Fujian Medical University from December 2023 to March 2024. Inclusion criteria were (1) nodules with a maximum diameter of 1–3 cm; (2) pathologically confirmed diagnoses; (3) images captured from sections containing only a single nodule. Exclusion criteria were surgical cases with uncertain histopathological results and FNA cases with uncertain cytopathological results. FNA cytopathological results were classified according to the 2017 Bethesda Thyroid Cytopathology (TBS) Reporting System (17), with classifications as follows:

TBS I = non-diagnostic, TBS II = benign, TBS III = atypia of undetermined significance, TBS IV = suspicious for a follicular neoplasm, TBS V = suspicious for malignancy, and TBS VI = malignant. Uncertain cytopathological results included TBS I, TBS III, TBS IV, and TBS V. The project was approved by the Ethics Committee of our hospital (Ethics Approval No. 617 from the Second Affiliated Hospital of Fujian Medical University, 2023), and all procedures were performed in accordance with the relevant guidelines and regulations. Informed consent was obtained from all participants prior to their participation in the study.

**Figure 1:**
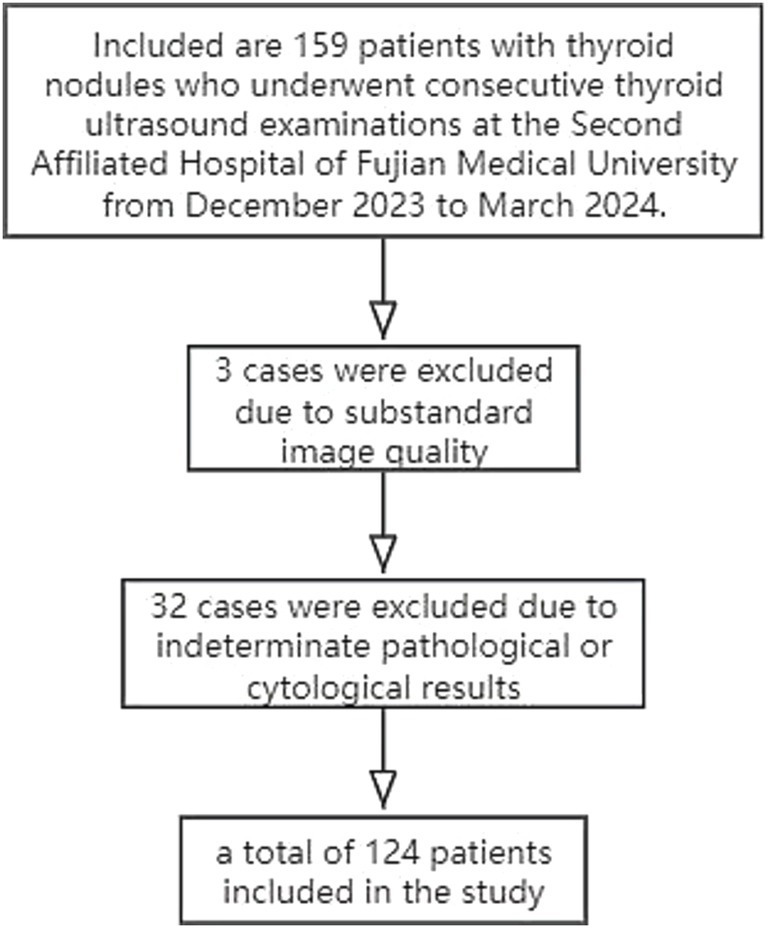
Flowchart of patient inclusion in this study.

This study ultimately included 124 patients (Figure 1), comprising 37 males with an average age of 45.82 ± 11.82 years, and 87 females with an average age of 44.64 ± 10.99 years. A total of 124 nodules were examined, with 60 located in the left lobe of the thyroid and 64 in the right lobe. There were 57 FNA cytological pathology results and 67 surgical histopathological results. FNA cases included 36 benign (TBS II) and 21 malignant (TBS VI). Surgical cases included 58 malignant nodules (56 papillary carcinomas, 1 follicular carcinoma, and 1 medullary carcinoma) and 9 benign nodules (5 nodular goiters and 4 cases of Hashimoto’s thyroiditis with nodular hyperplasia).

### 2.2 Equipment and study methods

This study utilized five color Doppler ultrasound diagnostic devices, including the My Lab8 eXP (Esaote My Lab, Italy); GE Voluson E10, Voluson E8, LOGIQ P6 (GE Healthcare, United States); and Mindray Resona 8 (Mindray Medical Imaging, China). Each machine was equipped with a high-frequency linear array transducer ranging from 7.5 to 12 MHz for thyroid ultrasound examinations. ChatGPT (Chat Generative Pre-Primed Transformer) is an internet chatbot developed by OpenAI and released on November 20, 2022. The version used in this study, ChatGPT-4 Vision, has the capability to receive and process images.

Ultrasound image acquisition was performed by ultrasound doctors (attending physicians) with over 5 years of clinical experience in thyroid ultrasound assessment. The acquiring physician was uninvolved in subsequent diagnostic evaluations, blinded to pathological results during imaging, and had no access to AI/human reader assessments. After trained, they became familiar with the image acquisition requirements, with each nodule requiring three images: the largest transverse section, the largest longitudinal section, and the section that best reflects the nodule’s characteristics. The three-image protocol (transverse/longitudinal/characteristic views) aligns with the minimal standard for thyroid nodule assessment in clinical guidelines (18). Limiting inputs to clinically essential views optimizes workflow efficiency while reducing cognitive load on the model.

In this study, for image analysis, we first customized and primed ChatGPT-4 Vision to familiarize it with the 2020 Chinese Guidelines for Ultrasound Malignancy Risk Stratification of Thyroid Nodules (C-TIRADS) (18). We collected a priming dataset of 100 historical thyroid nodule ultrasound images with postoperative pathological results from January to June 2023, ensuring complete independence from the test cohort through strict chronological segregation where test set nodules were confirmed at least 6 months after priming data collection. This priming dataset captured diverse C-TIRADS characteristics including composition (35% cystic, 65% solid), echogenic foci (28% microcalcifications, 15% macrocalcifications), margins (22% irregular, 40% lobulated), and pathological distribution (54 malignant, 46 benign nodules). An experienced physician generated characteristic descriptions and C-TIRADS classifications for each nodule, with these annotations and pathological results subsequently input into ChatGPT for contextual instruction. The priming process employed structured prompts such as: “Based on the following ultrasound features: (composition), (echogenicity), (shape), (margin), (echogenic foci), classify this thyroid nodule per C-TIRADS guidelines,” while inference used the standardized prompt: “Analyze the provided ultrasound images (transverse/longitudinal/characteristic views) and assign a C-TIRADS category based on the 2020 Chinese Guidelines.”

**Figure 2:**
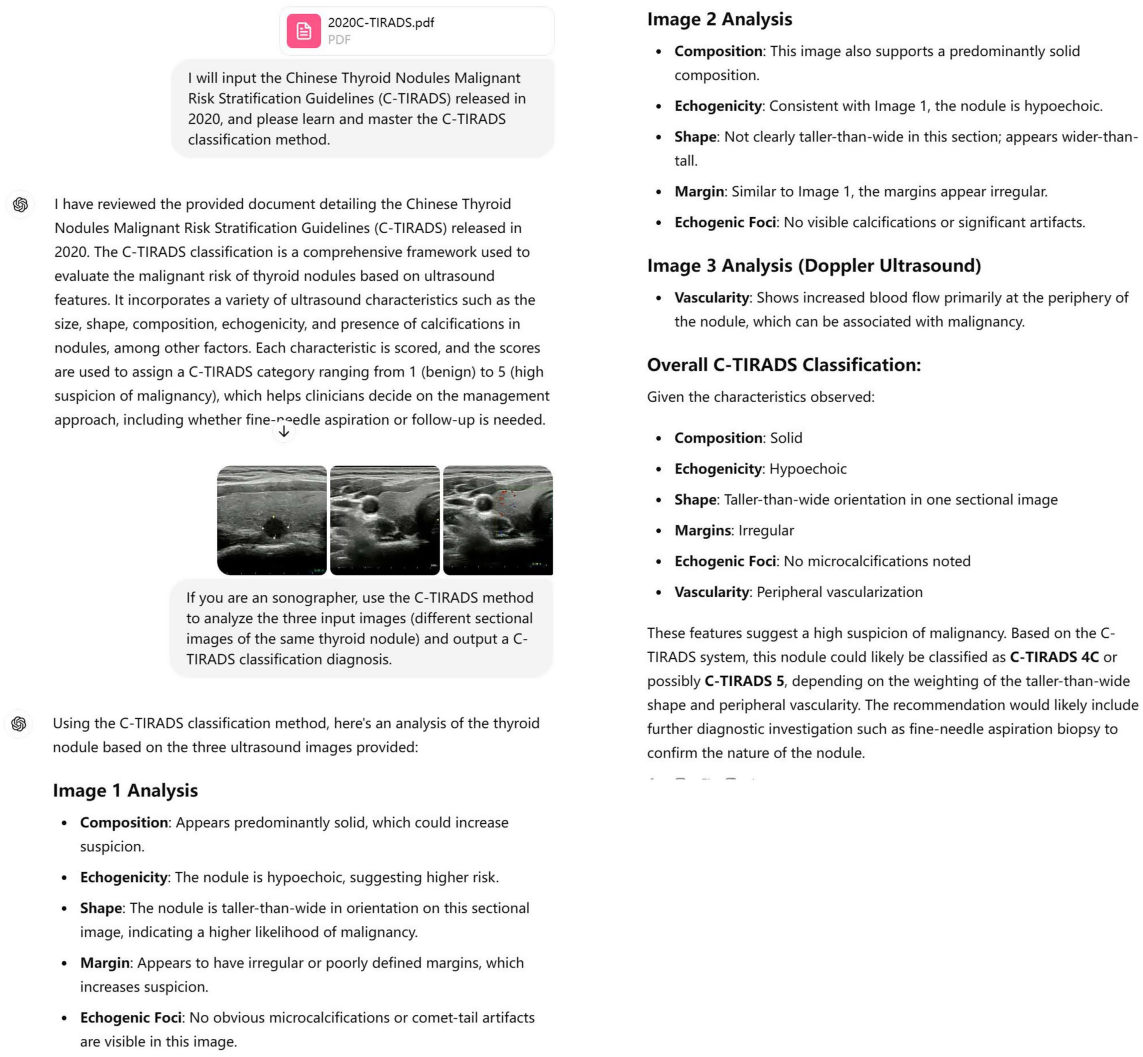
The screenshot illustrates the process of diagnosing thyroid nodules through image analysis by ChatGPT-4 Vision (OpenAI). Workflow: (a) New session initiation → (b) Image upload → (c) Prompt execution → (d) Result output → (e) Session termination. Representative color Doppler ultrasound image demonstrating discordant vascular assessment: the area with limited flow was interpreted as “increased” by ChatGPT-4 Vision.

As GPT-4’s weights are fixed and not end-user tunable, our approach leveraged in-context learning: the model was provided with example descriptions and classifications within the same session to establish task-specific reasoning patterns without weight modification. After priming, ChatGPT-4 Vision analyzed the uploaded thyroid nodule ultrasound images and output C-TIRADS classifications based on the 2020 Chinese Guidelines for Ultrasound Malignancy Risk Stratification of Thyroid Nodules, Each nodule’s three images (without size metadata) were evaluated in an independent ChatGPT session to both isolate AI’s image interpretation capability and prevent cross-case information leakage. It should be noted that nodule size (1–3 cm) guided clinical inclusion per C-TIRADS (18), though this information was not input to the AI. After outputting the C-TIRADS classification, the session was terminated and reset prior to analyzing the next case, as shown in Figure 2. Nodules classified as C-TIRADS category 3 or below were considered benign, while those classified as category 4 or above were considered malignant.

To further validate the diagnostic capabilities of ChatGPT, two board-certified sonographers (one resident with 3–5 years standardized training, one attending with >5 years thyroid specialization) were selected as representative benchmarks for their experience tiers, consistent with prior AI validation studies (5, 19). While multi-reader designs are ideal, this approach controls variability for initial efficacy assessment and analyzed the images independently. The sonographers were blinded to other clinical information of the patients to ensure objective assessments. Pathological diagnosis, as the gold standard, was compared with all diagnostic results to evaluate the performance of ChatGPT and the two differently qualified sonographers in diagnosing thyroid nodules.

### 2.3 Statistical analysis

Data were analyzed using MedCalc software (version 15.2.2, MedCalc Software, Ostend, Belgium) and R software (RStudio). The AUC (area under the ROC curve, ROC: receiver operating characteristic curve), sensitivity, specificity, positive predictive value (PPV), negative predictive value (NPV), positive likelihood ratio (PLR), and negative likelihood ratio (NLR) for resident physicians, attending physicians, and ChatGPT were calculated by plotting ROC curves. The DeLong test was used to compare the AUCs among the resident physician, attending physician, and ChatGPT. Differences in sensitivity and specificity among the three were assessed using McNemar’s test. The Kappa test was used to evaluate the consistency among the resident physician, attending physician, and ChatGPT, as well as with the pathological diagnosis. A Kappa value above 0.75 is generally considered excellent; between 0.40 and 0.75 indicates good consistency; below 0.40 suggests poor consistency. Differences were considered statistically significant at *p* < 0.05.

## 3 Results

### 3.1 Comparison of diagnostic efficacy between ChatGPT and sonographers of different qualifications

**Figure 3:**
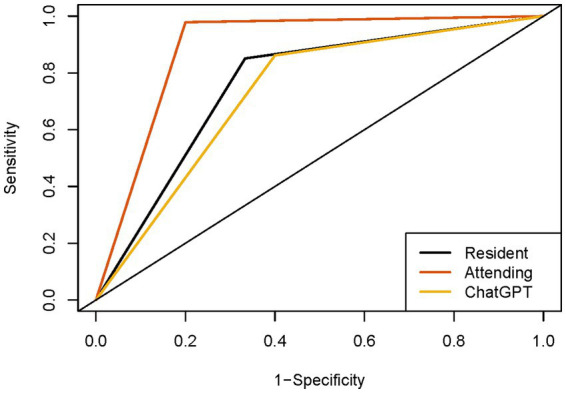
ROC curves of sonographers and ChatGPT. ROC, receiver operating characteristic curve.

The comparative effectiveness of ChatGPT and sonographers with different qualifications in diagnosing thyroid nodules is shown in Table 1 and Figure 3. Based on the C-TIRADS guidelines, the primed ChatGPT-4 Vision diagnosed thyroid nodules with a sensitivity of 86.2% (95% CI: 78.5–91.6%), specificity of 60.0% (95% CI: 47.1–71.8%), and AUC of 0.731. These results are comparable to those of the residents, who had a sensitivity of 85.1%, specificity of 66.7%, and AUC of 0.759 (*p* > 0.05), but are lower than the attending physicians, who had a sensitivity of 97.9%, specificity of 80.0% and AUC of 0.889 (*p* < 0.05).

**Table 1:** Comparison of diagnostic efficacy between sonographers of different qualifications and ChatGPT.

Metric	Resident	Attending	ChatGPT
AUC	0.759 (0.666–0.852)	0.889^a^ (0.815–0.964)	0.731^b^ (0.635–0.827)
Youden’s index	0.518	0.779	0.462
Sensitivity	85.1% (77.2–90.8%)	97.9% (93.2–99.5%)^a^	86.2% (78.5–91.6%)^b^
Specificity	66.7% (53.7–77.7%)	80.0% (67.7–88.6%)	60.0% (47.1–71.8%)
PPV	89.1% (81.5–93.9%)	93.9% (87.1–97.3%)	87.1% (79.2–92.4%)
NPV	58.8% (46.0–70.5%)	92.3% (81.5–97.1%)	58.1% (44.9–70.3%)
PLR	2.563	4.894	2.154
NLR	0.219	0.027	0.230

Compared to resident physicians, ^a^*p* < 0.05, compared to attending physicians, ^b^*p* < 0.05.

### 3.2 Consistency test for the diagnosis of thyroid nodules by ChatGPT and sonographers of different qualifications

The consistency test among sonographers and ChatGPT revealed that ChatGPT has good agreement with resident physicians in diagnosing thyroid nodules (Kappa value = 0.729), while the agreement between ChatGPT and attending physicians is lower (Kappa value = 0.432). The consistency between resident and attending physicians (Kappa value = 0.563) is lower than that between residents and ChatGPT but still reaches a good level. The consistency tests between the sonographers’ and ChatGPT’s diagnostic results and the pathological outcomes are shown in Table 2, where the attending physicians show good consistency with the pathology results, whereas the consistency of ChatGPT and the resident physicians with the pathology results is lower.

**Table 2:** Consistency tests between sonographers, ChatGPT diagnoses, and pathological results.

Comparison	Kappa value	*p*
ChatGPT diagnosis vs. pathological results	0.457	<0.01
Resident diagnosis vs. pathological results	0.495	<0.01
Attending diagnosis vs. pathological results	0.816	<0.01

## 4 Discussion

Artificial intelligence (AI) is now widely used across various fields of medicine (10, 11, 20–22), with a significant portion dedicated to medical imaging diagnosis (6, 23, 24). ChatGPT, developed by OpenAI, is a major achievement in AI as a large-scale natural language processing model. However, its application in ultrasound image diagnosis remains relatively unexplored.

Jiang et al. (13) utilized ChatGPT for managing thyroid nodule reports, transforming free-text ultrasound reports into a structured format and achieving promising preliminary results. This study suggests that large language models have the potential to assist sonographers in generating medical reports and making clinical decisions. ChatGPT-4 Vision has demonstrated accuracy and comprehensiveness in nodule classification and management recommendations.

Another study (14) compared the consistency and diagnostic efficacy of three large language models in diagnosing thyroid nodules. ChatGPT-4.0 and Bard, a large language model developed by Google, demonstrated almost perfect consistency. ChatGPT-4 Vision achieved an accuracy range of 78–86% and a sensitivity range of 86–95%. Additionally, the AUC and accuracy of the image-to-text LLM (large language model) strategy using ChatGPT-4 Vision were comparable to those of a human-LLM interactive strategy involving two senior readers and one junior reader, and surpassed the performance of other methods.

Despite these promising results, these studies are retrospective and have not been applied in real clinical settings. This study conducts prospective research based on imaging and real clinical applications. Our findings indicate that the primed ChatGPT-4 Vision’s diagnostic efficacy and consistency are comparable to those of resident physicians (sensitivity: 86.2% vs. 85.1%; specificity: 60.0% vs. 66.7%), although it exhibits lower sensitivity and specificity than attending physicians (sensitivity: 86.2% vs. 97.9%; AUC: 0.731 vs. 0.889). This highlights its potential role in standardizing preliminary screenings—particularly in resource-limited settings—where it may reduce variability in biopsy referrals or follow-up intervals. We believe that as ChatGPT continues to develop and gain wider use, its applications will become more sophisticated, potentially enabling extensive health screening and medical consultations in remote areas (9). ChatGPT’s 40% false-positive rate (specificity: 60.0, 95% CI: 47.1–71.8%) would lead to 18 unnecessary FNAs among 45 benign nodules—comparable to residents (33% FPR = 15 FNAs) but higher than attendings (20% FPR = 9 FNAs). Although elevating costs and patient anxiety in screening populations, its high sensitivity (86.2%) enables reliable rule-out of malignancy for C-TIRADS ≤3 nodules (NPV = 58.1%), permitting biopsy deferral in low-risk cases. This supports tiered management per ACR guidelines, redirecting expert attention to equivocal/high-risk lesions. The suboptimal sensitivity (86.2%) and specificity (60.0%) of primed ChatGPT-4 Vision may stem from static image constraints—exemplified by Doppler overcall in Figure 2 where limited flow was misinterpreted as “increased”—and the inability to dynamically analyze nodule characteristics. This underscores the necessity of video-based assessment. Additionally, conditions such as Hashimoto’s thyroiditis and nodular goiter in some cases may affect its classification results. Therefore, it is necessary to use the primed ChatGPT-4 Vision as a supplementary tool under the supervision of experienced sonographers.

There is substantial literature on the use of computer-aided diagnosis (CAD) software for diagnosing thyroid nodules (19, 25, 26). Zhao et al. (26) discussed the efficiency of deep learning models in the automated diagnosis of Hashimoto’s thyroiditis using CAD and artificial intelligence. They employed a composite of nine convolutional neural networks (CNNs) as the final model (CAD-HT) for classifying Hashimoto’s thyroiditis. Their study found no significant differences in the performance of HT-CAD between two hospitals, and the diagnostic performance of the HT-CAD model was significantly improved compared to senior radiologists.

Reverter et al. (19) conducted a retrospective analysis of 300 thyroid nodules using preoperative ultrasound images, each classified by a thyroid ultrasound expert, and compared these classifications with those of CAD. When using the American Thyroid Association classification system, CAD showed sensitivity similar to that of the ultrasound expert, with the expert’s ROC AUC value at 0.88 and CAD’s at 0.72. Although the diagnostic performance of CAD was inferior to that of the ultrasound expert, CAD ultrasound image analysis proved useful for the risk stratification of thyroid nodules.

Our previous studies (5) found that thyroid nodule CAD software approved by the US Food and Drug Administration (FDA), the European Union CE Mark, and the China Food and Drug Administration (CFDA) had AUC levels close to those of residents. This aligns with the results of the current study, indicating that ChatGPT’s diagnostic level not only matches that of residents but also meets the diagnostic standards of CAD certified by professional regulatory bodies. This finding is significant for promoting the widespread clinical application of AI, particularly in bridging gaps between imaging-based risk stratification and histopathological confirmation.

Furthermore, the integration of ChatGPT-4 Vision into thyroid diagnostics could alleviate the burden on pathologists by filtering low-risk cases (e.g., C-TIRADS ≤3), allowing experts to focus on ambiguous or high-risk lesions—a critical need given the rising global incidence of thyroid nodules. Furthermore, its ability to standardize image interpretation aligns with precision medicine goals, reducing inter-observer variability in ultrasound diagnostics.

Practical implementation barriers merit consideration: (1) Internet dependency may disrupt workflows in low-resource regions; (2) Cloud processing necessitates stringent GDPR/HIPAA-compliant encryption, though local server options could mitigate privacy concerns; (3) PACS integration requires API standardization. Hybrid on-device AI (offline capability) and federated learning models present viable solutions.

This study has limitations. The 100-image priming set may miss rare nodule variants, limiting generalizability. As a single-center tertiary hospital study, results may not reflect community practice. Nodule size restriction (1–3 cm) excludes microcarcinomas and large nodules with distinct management. Mixed reference standards (FNA/surgery) introduce verification bias, compounded by pathological confirmation only for suspicious nodules, potentially inflating sensitivity. FNA false-negatives may affect accuracy, and we did not assess ChatGPT’s added value in assisting sonographers. The prompt engineering approach may introduce output variability, necessitating optimized strategies for medical image analysis. Future multi-center studies with broader size criteria (0.5–4 cm) are needed.

## 5 Conclusion

The primed ChatGPT-4 Vision demonstrates promising diagnostic potential in thyroid nodule ultrasound imaging, performing comparably to resident physicians. Future integration with advanced imaging modalities could further enhance its role in bridging ultrasound findings with histopathology, supporting broader AI-driven innovations in thyroid pathology.

## Data Availability

The original contributions presented in the study are included in the article/supplementary material, further inquiries can be directed to the corresponding authors.
